# Spatial Attention Changes Excitability of Human Visual Cortex to Direct Stimulation

**DOI:** 10.1016/j.cub.2006.11.063

**Published:** 2007-01-23

**Authors:** Sven Bestmann, Christian C. Ruff, Colin Blakemore, Jon Driver, Kai V. Thilo

**Affiliations:** 1 Institute of Neurology, Wellcome Department of Imaging Neuroscience, University College London, London WC1N 3BG, United Kingdom; 2 Institute of Neurology, Sobell Department of Motor Neuroscience and Movement Disorders, University College London, London WC1N 3BG , United Kingdom; 3 Institute of Cognitive Neuroscience, University College London, London WC1N 3AR, United Kingdom; 4 Department of Physiology, Anatomy and Genetics, University of Oxford, Oxford OX1 3PT, United Kingdom; 5 Department of Psychology, Royal Holloway, University of London, Egham, Surrey TW20 0EX, United Kingdom

**Keywords:** SYSNEURO

## Abstract

Conscious perception depends not only on sensory input, but also on attention 1, 2. Recent studies in monkeys 3, 4, 5, 6 and humans 7, 8, 9, 10, 11, 12 suggest that influences of spatial attention on visual awareness may reflect top-down influences on excitability of visual cortex. Here we tested this specifically, by providing direct input into human visual cortex via cortical transcranial magnetic stimulation (TMS) to produce illusory visual percepts, called phosphenes. We found that a lower TMS intensity was needed to elicit a conscious phosphene when its apparent spatial location was attended, rather than unattended. Our results indicate that spatial attention can enhance visual-cortex excitability, and visual awareness, even when sensory signals from the eye via the thalamic pathway are bypassed.

## Results and Discussion

Transcranial magnetic stimulation (TMS) of human visual cortex, at an intensity above a distinct threshold, induces illusory visual perceptions called phosphenes 13, 14. It is thought that phosphenes originate from early visual cortex (V1/V2) 13, 14, 15, 16 and depend on the integrity [14] and excitability 17, 18 of the occipital region. The perceived phosphene lies within the visual hemifield contralateral to the stimulated cortical hemisphere, at a location reflecting the retinotopic organization of visual cortex. For example, TMS of superior right occipital cortex elicits a phosphene in the lower-left quadrant of the visual field.

This spatial specificity enabled us to use phosphene perception as a probe measure of cortical excitability, in two related experiments, under conditions of transient or sustained spatial attention. This allowed us to test directly whether spatial attention influences excitability of visual cortex, as revealed by phosphenes induced by TMS of visual cortex, which bypasses the retinogeniculate pathway.

Attention was covertly (without displacement of gaze) directed toward a particular location (left or right) during a task involving real visual stimuli. But on some trials, instead of real visual stimuli, TMS was applied, which (when sufficiently intense) produced a phosphene, either within the attended location or in the symmetric location in the opposite hemifield. This allowed us to measure whether conscious perception of TMS-induced phosphenes can be influenced by spatial attention. The TMS intensity needed to induce a conscious phosphene (i.e., the phosphene threshold [PT]) is widely held to provide a measure of visual-cortex excitability 17, 18. Hence, if we were to find that PTs are affected by spatial attention, this would imply a direct effect of attention on visual-cortex excitability as well as the corresponding awareness.

At or above threshold intensity, TMS phosphenes were experienced as small, brief, illusory white flashes of light, clearly localized at a particular position in the contralateral hemifield 14, 19, 20. PTs were determined by adjusting TMS intensity as a function of the participant's response, according to an adaptive converging-staircase algorithm 19, 21. TMS trials were randomly interleaved with trials in which real visual stimuli were presented for judgement at the same eccentricity in space as the possible TMS phosphene. Variation of the side to which covert attention was directed for the real visual task allowed measurement of PTs with and without spatially congruent attention. Importantly, we never gave TMS simultaneously with real visual stimuli here, unlike in some other studies that used TMS to suppress perception of visual stimuli 22, 23, 24. Instead, trial types were interleaved unpredictably such that TMS inputs *substituted* for real visual stimulation on some trials.

**Figure 1:**
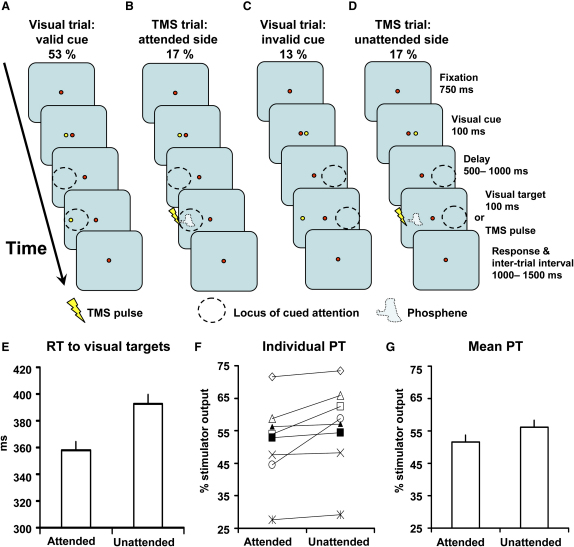
Transient Direction of Spatial Attention Affects Thresholds for TMS Phosphenes at Attended versus Unattended Locations (A–D) Cartoons of event sequences, with timing, for the four randomly interleaved trial types. A symbolic cue (amber LED 1° left or right of central fixation) instructed the observer to shift spatial attention covertly to the left (A and B) or the right (C and D). In visual trials ([A and C]: two-thirds of all trials), a peripheral target LED was illuminated, either on the side to which attention had been cued (validly cued, e.g., [A]), or on the other side (invalidly cued, e.g., [C]). Participants pressed a single response button with their right hand as soon as they saw the target LED. One-third of all trials were TMS probe trials (B and D), in which a TMS pulse was delivered 400 ms after cue offset, rather than a visual target. For half of these trials, the resultant phosphene (when experienced) appeared on the attended side, in the same part of the field in which visual stimuli were expected (e.g., [B]). For the other half (e.g., [D]), any phosphene was on the unattended side. (E) Reaction time (RT) to the visual target was significantly faster (Z = 2.52, p = 0.012) when its position was validly cued, confirming that spatial attention was directed as instructed. Standard error of the mean (SEM) RT difference between conditions is also shown. (F) Individual phosphene thresholds (PT), expressed as the percentage of maximum TMS output, plotted separately for phosphenes on the attended or unattended sides for each participant. PTs were significantly higher for the unattended location (Z = 2.52, p = 0.012). Data have been pooled across left or right hemifields because there were no differences between the two sides per se. (G) Group mean PTs and SEM differences between attended and unattended conditions.

In Experiment 1, participants maintained gaze on a central point throughout, while covertly directing spatial attention toward either the left or the right hemifield on each trial, according to a randomly determined prior cue signal that preceded the presentation of the target (Figures 1A–1D). A briefly illuminated LED (100 ms), on one side or other of the fixation point acted as an attentional cue, indicating the side on which a peripheral visual target was likely to appear for that trial. On two-thirds of trials, after a variable interval, a real, suprathreshold visual target then appeared briefly, and participants had to press a button as quickly as possible. For 80% of these “visual” trials, the external target was on the side to which attention had been directed (validly cued); for an unpredictable 20%, the target appeared in the opposite hemifield (invalidly cued). Reaction times for detection of visual targets were faster to validly cued than to invalidly cued targets (358 ± 21 ms versus 393 ± 23 ms (mean ± SEM), Z = 2.52, p = 0.012; see Figure 1E). This confirms that spatial attention was indeed directed to the cued location for the external task.

On one-third of the trials, randomly interleaved with the valid and invalid visual trials, no external visual target appeared. Instead, a single TMS pulse was applied to one side or the other of the occiput, ipsilateral or contralateral to the hemifield that had been cued for attention. On these TMS trials, participants reported verbally whether they experienced a visual phosphene or not. Our critical finding was that PTs were significantly lower for trials on which the phosphene appeared in the hemifield that had been cued for attention (51.6% of maximum stimulator output [MSO], compared with 56.2% MSO for the other side; Z = 2.52, p = 0.012; see Figures 1F and 1G). This indicates that transiently cued spatial attention enhances visual awareness, even for direct TMS of visual cortex, which bypasses the pathway from the eye, hence precluding feedforward thalamic gating of initial afferent sensory input.

**Figure 2:**
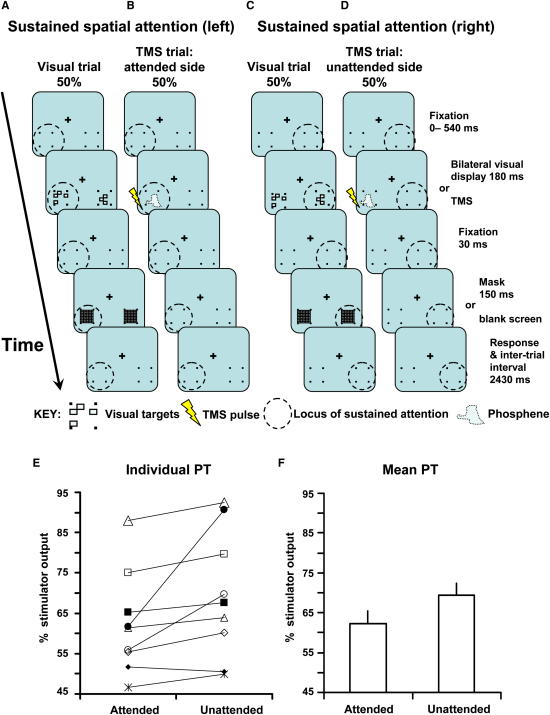
Thresholds for TMS Phosphenes Are Affected by Spatial Attention that Is Sustained throughout a Block As in Figure 1, panels (A)–(D) show event sequences for four different types of trial. During central fixation, covert spatial attention was directed continuously toward either the left (A and B) or the right (C and D) lower quadrant throughout each block (now avoiding presentation of visual stimuli at unexpected locations). Within each block, 50% of trials were visual (A and C) and 50% were TMS (B and D), randomly interleaved. The visual stimuli were groups of one to four gray rectangles on either side of 1° each, bilaterally presented within a defined square target region in each hemifield, below the horizontal meridian, and followed by a masking checkerboard covering the target area (8° square). Participants had to report via key-press the number of rectangles in the target area on the attended side only. On TMS trials, with spatial attention sustained toward one side or the other for the external task, a TMS pulse was applied to the right side of the occiput, and the observer had to indicate whether or not a phosphene was experienced. (E) Individual PT values. In eight out of nine subjects, the PT was lower in blocks where spatial attention was continuously directed toward the area of the phosphene. (F) The group mean PT was significantly lower for the attended quadrant than for the unattended (Z = 2.55, p = 0.011). SEM differences between attended and unattended conditions is also shown.

It is conceivable that the heightened thresholds for the unattended noncued hemifield in Experiment 1 were due, at least partly, to the fact that stimuli on the noncued side (real visual or TMS-induced phosphene) were less frequent overall. Experiment 2 eliminated this potential “surprise” factor and thereby possible differences in criteria for reports associated with different probabilities of event occurrence on one side versus the other. We then tested PTs under conditions of sustained rather than transient spatial attention. The particular side on which the external target had to be detected was now constant throughout each block. For each block of 96 trials, participants were instructed to maintain their covert attention continuously on the lower quadrant of one particular hemifield while they retained central fixation (confirmed with eye-tracking). Visual stimuli, which were presented simultaneously and bilaterally (see Figures 2A–2D), consisted of a variable number (one to four) of target rectangles, independently selected on the two sides for each trial. Participants had to indicate, via button-press, the number of such rectangles on the attended side only.

On a critical 50% of randomly interleaved trials in each block (with attention sustained to one quadrant for the external visual task), a TMS pulse was delivered to the right side of the occiput *instead of* visual stimulation. Observers had to report whether they experienced a phosphene (which now fell within the target area used for real visual stimuli in the left hemifield; see Figures 2A–2D). As TMS was now applied to only one hemisphere, more PT measurements could be obtained than in Experiment 1, allowing fitting of full psychometric functions to the response data (see Experimental Procedures and Figure S1 in the Supplemental Data available online).

In half of the blocks, attention was directed constantly to the left (the side of possible phosphene appearance) for the rectangle-counting task; in the other half, it was directed to the right. The critical finding was again that PTs were significantly lower when spatial attention was congruent with the side of the phosphene (62.3% MSO, compared with 69.4% MSO for the other side; Z = 2.55, p = 0.011; Figures 2E and 2F). Moreover, whereas these thresholds differed reliably as a function of attention, the slopes of the underlying psychometric functions did not (see Experimental Procedures and Figure S1). This is consistent with a genuine increase in cortical excitability as a result of spatial attention, rather than a criterion shift.

Recent demonstrations that attention can modulate activity in the human lateral geniculate nucleus (LGN) 25, 26 reopened the long-standing question of whether thalamic gating of afferent inputs may contribute to, or even be necessary for, effects of spatial attention on visually evoked activity in striate 5, 6, 7, 8 and extrastriate cortex 6, 7, 9. Initial modulation of feedforward signals at the LGN might conceivably be a necessary prerequisite for effects of spatial attention to be propagated to and enhanced at subsequent cortical areas, with possible amplification at successive stages [27]. On the other hand, many authors have previously suggested or implied that top-down influences on early visual cortex 10, 11, 12, 28, 29, 30, 31, producing so-called baseline shifts 32, 33, 34, might arise without thalamic mediation. Typically such arguments against a critical role for the thalamus have been based on indirect evidence, from the relative timing of components in event-related potentials (ERPs) or from the absence of observable attentional effects in neuroimaging of the thalamus (which, in some cases, might have been attributable to use of relatively low-resolution imaging in some cases). By contrast, the present experiments were able to test the issue directly by using cortical stimulation and demonstrate a positive effect that *cannot* be attributed to thalamic gating of initial afferents.

This new evidence confirms prior proposals that top-down attentional influences (e.g., from frontal or parietal cortex) change activity and excitability of visual cortex itself (e.g., 2, 8, 28, 29). It is also compatible with studies in which the relative timing of ERP components was taken to indicate that attentional modulation of visual cortex is more likely to reflect top-down feedback than feedforward thalamic gating 10, 11, 12, 28, 29, 30.

What, then, should we make of the clear evidence that visual activation of the LGN itself can be influenced by spatial attention 25, 26? We suggest that this may reflect recursive corticothalamic interactions [35], in line with the time at which spatial attention appears to influence early visual-cortex excitability 10, 11, 12, 28, 30. Although during “normal” visual perception such recursive processing may indeed contribute to spatial attention, our results show that thalamic gating of initial afferents is not a necessary precondition for attentional modulation of cortical excitability.

Our results further demonstrate that attention can act directly on the cortex, enhancing excitability of neural populations that process the attended region in visual space. This appears to be a very general neural mechanism that can affect the very different inputs and featural properties of our two types of stimuli (i.e., cortical TMS as well as real optical stimulation) and that might apply across different degrees of attentional load [36], as for Experiments 1 and 2 here. In conclusion, our study shows directly that spatial attention enhances excitability in visual cortex to facilitate visual awareness, even when retinal stimulation, retinogeniculate conduction of sensory signals, and thalamic gating of feedforward afferents are ruled out.

## Experimental Procedures

In both experiments, participants wore earplugs and headphones, and stable viewing and head position were ensured with a chinrest and nose-bridge. All participants had normal or corrected visual acuity and reported no history of neuropsychiatric illness or epilepsy. All gave informed consent in accord with local ethics approval.

### Stimuli and Procedure

There were eight participants (aged 24–29 yr, mean 26.5 yr, 4 females) for Experiment 1. Each observer sat in a darkened room, 40 cm from a central fixation target (red LED), with the TMS positioned over one side of the occiput. Trials were initiated by button-press. A small target light (amber LED) was positioned at the center of the region of the visual hemifield contralateral to the TMS coil that corresponded to the spatial location of the phosphene produced by suprathreshold TMS (individual range, 10°–23° lateral eccentricity; mean 11.54° ± standard deviation [SD] 6.39°). An identical target light was set symmetrically at the same eccentricity in the other hemifield. The task for external visual targets was to report their onset via speeded button-press. See Figure 1 for the temporal sequence of events in different trial types, with timing information.

On TMS trials, which were evident to the participants because of the audible click produced by the coil, they had to report verbally whether or not they had perceived a phosphene. Responses were entered into a PC, and TMS output intensity was automatically adjusted according to the modified binary search (MOBS) adaptive-converging algorithm [21]. The button for external visual targets was inappropriately pressed on 8% ± 1.9% (mean ± SD) of TMS trials. The exact number of TMS trials per subject depended on how quickly the MOBS algorithm converged on to a PT (mean 32 ± SD 9 TMS trials). PTs were determined separately for trials in which attention had been cued to the locus of phosphene appearance (see Figure 1B) and those in which the cue had directed attention to the opposite spatial location (Figure 1D). After PTs had been determined for TMS stimulation of one cortical hemisphere, the session was repeated with TMS applied to the other hemisphere, to elicit phosphenes in the opposite hemifield. The LEDs were again symmetrically positioned to match the location of the TMS phosphene. The order in which the hemispheres were stimulated was counterbalanced across participants. The sequence of trials was randomized across all trial types. In total, four phosphene thresholds were determined via MOBS for each participant: for left and right hemifields, with each either validly or invalidly cued, respectively.

In Experiment 2, eleven different participants (aged 19–31 yr, mean 23.5 yr, 6 females) performed a *sustained* spatial-attention task. They had been selected from a sample of 27 participants for reliably perceiving phosphenes in a well-circumscribed location, with eyes open, at 90% of TMS stimulator output or less (see [37]). Two participants were excluded from subsequent analysis because of excessive eye movement (see Supplemental Data).

Visual stimuli were presented in a darkened room on a 21 inch computer screen, refreshing at 60 Hz, at a viewing distance of 45 cm. A signal was presented at screen center for 2 s prior to the start of each block, to instruct the observer to direct attention covertly to one side for the whole of the block. The corners of each target area were demarcated by four small, gray marker dots, 2 pixels square and visible throughout the experiment, in order to help the observer sustain spatial attention on the relevant region for the visual task (see Supplemental Data). The target areas (each 8° square) were centered 8° below the horizontal meridian, at an eccentricity of 19° in the lower quadrants of both hemifields.

In visual trials (see Figures 2A and 2C), participants had to report the number of target rectangles in the attended quadrant only, by pressing one of four keys with digits 2 to 5 of the right hand. Target stimuli were presented for 180 ms, between 0 and 540 ms after trial initiation. Visual stimulation was bilateral, with the number of rectangles randomly selected on each side, but participants only had to judge the attended side. They had to respond within 2430 ms after presentation of the masking checkerboard. Apart from this, there was no emphasis on speed for this difficult discrimination (response latency was similar for all trial types: left correct, 1299 ms; left incorrect 1309 ms; right correct, 1285 ms; and right incorrect, 1277 ms; Friedman Test, χ^2^_(3)_ = 1.93; p = 0.57). On TMS trials (see Figure 2B and 2D) subjects reported the presence or absence of a phosphene via button-press of the left index or middle finger, respectively. No emphasis was given to the speed of response.

TMS intensities were again varied from trial to trial by using MOBS 19, 21, but whenever MOBS converged prior to the end of a block the procedure was started again, in order to present as many phosphene stimuli as possible throughout a block. The number of MOBS convergences per subject did not differ between experimental conditions (attend right, mean 7.00 ± SD 1.34; attend left, mean 7.18 ± SD 1.66). Given the larger dataset in Experiment 2, PTs could now be determined by fitting full Weibull psychometric functions [38] to the raw data as measured with MOBS, by using the MATLAB toolbox psignifit (see http://bootstrap-software.org/psignifit). This allowed assessment not only of PTs, but also of the slopes of the underlying psychometric functions (see Results and Discussion and Figure S1). However, note that the PTs determined by psychometric curve fits for Experiment 2 were highly correlated (r = 0.93 across all conditions, p < 0.001) with PTs when determined by MOBS convergence as in Experiment 1 (demonstrating that both methods are equally valid for our purposes). Importantly, the curve fits confirmed that the slopes of the psychometric functions in Experiment 2 did not differ between attended and unattended sides (Z = 0.53, p = 0.60), further supporting our finding of significant differences in threshold.

Each participant performed one practice block of 96 trials. The order of experimental blocks (two blocks each of leftward or rightward attention) and trial types was randomized. After two blocks, a break of approximately 5 min was given.

Subjects were repeatedly instructed to avoid eye movements, as confirmed by eye-tracking (see Supplemental Data). The Z values given for comparisons of attended versus unattended conditions in the main text and figure legends were derived from Wilcoxon signed-ranks comparisons.

### Transcranial Magnetic Stimulation

TMS was applied by using a 70 mm figure-of-eight coil (Super Rapid, Magstim, Dyfed, Wales, UK). A two-joint holder was used to place the coil to one side or other of the occiput, approximately 4 cm above the inion, at a laterality for which phosphenes were reliably reported [15]. The initial rising phase of the induced biphasic current (∼250 μs duration) had a temporomedial orientation, optimal for inducing visual phosphenes [14].

In Experiment 1, the location of the real visual targets (LEDs) was matched in eccentricity to the center of each individual's phosphene locus, reported before the collection of experimental data. In Experiment 2, the TMS coil was held in place as in Experiment 1, but with the stimulation site, on the right side of the occiput, chosen to maximize spatial congruence of phosphenes with the fixed target area for external visual stimuli in the lower-left quadrant on the computer screen. Note that TMS-evoked phosphenes are more readily elicited for lower quadrants of the visual field [15].

TMS output intensity was varied according to the MOBS algorithm 19, 21. The upper and lower boundaries were 100% and 0% of TMS output, respectively. The initial presentation was midway between these boundaries. The termination criteria were a maximum of five reversals and a maximum last step size of 5% of the initial TMS output range. Note that in this study, no visual stimuli were presented at the same time as TMS (unlike 22, 23, 24) because PTs are known to change with visual input (e.g., as a function of its luminance contrast [20]). Indeed, this is one of the reasons why PTs are widely considered to reflect excitability of visual cortex (just as TMS thresholds for induced movements reflect excitability of motor cortex [39]). TMS application produces a “click” sound, but this was equivalent across the different conditions of visual attention here, so it could not have confounded our results.

## Supplemental Data

Document S1. Experimental Procedures and One Figure
