# Supplementary material for: Spatial Attention Changes Excitability of Human Visual Cortex to Direct Stimulation
Source: Curr Biol. 2007 Jan 23;17(2):134–9. doi: 10.1016/j.cub.2006.11.063 (PMC1815217; doi:10.1016/j.cub.2006.11.063)
Supplement: Document S1. Experimental Procedures and One Figure [file mmc1.pdf]

Supplemental Data

Spatial Attention Changes Excitability of Human Visual Cortex to Direct Stimulation

Sven Bestmann, Christian C. Ruff, Colin Blakemore, Jon Driver, and Kai V. Thilo

Supplemental Experimental Procedures

Experiment 2

On the basis of pilot measurements, exposure durations for the visual stimuli were selected to titrate counting performance for attended stimuli to ~60% correct—clearly above chance (25%), but far from saturation—to provide a demanding spatial-attention task. In the experiment, accuracy was indeed around 60% (left, mean 61.28 ± SEM 12.89%; right, mean 59.08 ± SEM 11.27%). Further pilot work demonstrated that this level of performance required selective attention to one side (i.e., when observers were required to judge the number of targets for *both* sides on each trial, accuracy dropped substantially).

Eye Monitoring

Horizontal and vertical eye-position were monitored continuously by using an infrared ASL 601 Remote Optics Eyetracker (ASL, Applied Science Laboratories, Bedford, Massachusetts; 50 Hz sampling frequency). Eye-position data were analyzed with the open-source toolbox ILAB [S1]. Eye blinks were identified and removed from the eye recordings prior to further analysis. Seven hundred millisecond sweeps of horizontal and vertical eye movements were then analyzed, commencing 350 ms prior to target presentation or TMS, and temporally filtered by convolution with a Gaussian (five sampling points full-width at half maximum [FWHM]). Saccades (eye velocity > 30 deg/s) or significant ocular drift (>1° per 100 ms epoch) were identified by using previously published criteria [S2, S3]. Any trial on which gaze deviated, by these criteria, outside the 1° square window around the fixation point was rejected. On average, 7.47% (interparticipant range 3.79%–13.27%) of trials were discarded because of such loss of central fixation.

Supplemental References

S1. Gitelman, D.R. (2002). ILAB: A program for postexperimental eye movement analysis. Behav. Res. Methods Instrum. Comput. *34*, 605–612.

S2. Fischer, B., Biscaldi, M., and Otto, P. (1993). Saccadic eye movements of dyslexic adult subjects. Neuropsychologia *31*, 887–906.

S3. Fischer, B., Weber, H., Biscaldi, M., Aiple, F., Otto, P., and Stuhr, V. (1993). Separate populations of visually guided saccades in humans: Reaction times and amplitudes. Exp. Brain Res. *92*, 528–541.

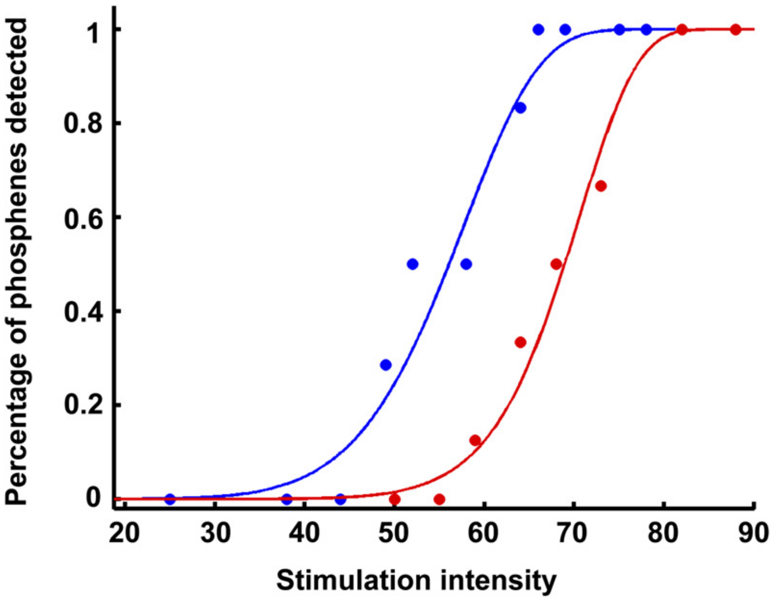

Figure S1. Psychometric Curves for an Illustrative Participant

Psychometric curves fitted to the phosphene report data for the attended (blue) and unattended (red) hemifield, for an illustrative participant. The curve was fitted by a Weibull psychometric function by using the MATLAB toolbox psignifit (see http://bootstrap-software.org/psignifit). The dotted lines indicate the TMS intensity at which phosphenes were perceived in half of the trials. Note the lateral shift but preserved slope of the psychometric curve, indicating a genuine threshold change for attended versus unattended phosphenes, rather than a shift of criterion. PTs determined by psychometric curve fits for Experiment 2 were highly correlated (r = 0.93 across all conditions, p < 0.001) with PTs when determined by MOBS convergence as in Experiment 1.
